# Preventive effects of systemic *Pistacia eurycarpa Yalt*. administration on alveolar bone loss and oxidative stress in rats with experimental periodontitis

**DOI:** 10.1590/1678-7757-2023-0344

**Published:** 2024-01-29

**Authors:** Mustafa Atalay, Mustafa Özay Uslu, Mehmet Sina İçen, Nuray Üremiş, Yusuf Türköz

**Affiliations:** 1 75th Year Oral and Dental Health Hospital Ministry of Health Ankara Turkey Ministry of Health, 75^th^ Year Oral and Dental Health Hospital, Ankara, Turkey; 2 Alanya Alaaddin Keykubat University Faculty of Dentistry Department of Periodontology Antalya Turkey Alanya Alaaddin Keykubat University, Faculty of Dentistry, Department of Periodontology, Antalya, Turkey.; 3 Inonu University Faculty of Pharmacy Department of Pharmacognosy Malatya Turkey Assistant Prof. Dr. Mehmet Sina İçen, Inonu University, Faculty of Pharmacy, Department of Pharmacognosy, Malatya, Turkey.; 4 Inonu University Faculty of Medicine Department of Medical Biochemistry Malatya Turkey Ph.D Nuray Üremiş, Inonu University, Faculty of Medicine, Department of Medical Biochemistry, Malatya, Turkey.; 5 Inonu University Faculty of Medicine Department of Medical Biochemistry Malatya Turkey Prof. Dr. Yusuf Türköz, Inonu University, Faculty of Medicine, Department of Medical Biochemistry, Malatya, Turkey.

**Keywords:** Alveolar bone loss, Periodontitis, Plant extracts, Oxidative stress

## Abstract

**Objective:**

This study aimed to investigate the effects of systemic administration of *P. eurycarpa Yalt*. plant extract on alveolar bone loss and oxidative stress biomarkers in gingival tissue in a rat model of experimental periodontitis.

**Methodology:**

32 male Wistar albino rats, weighing 200–250 g, were divided into four groups (n=8): Healthy control (HC), Experimental periodontitis control (EPC), Experimental periodontitis 400 mg/kg (EP400), Experimental periodontitis 800 mg/kg (EP800). Experimental periodontitis was induced using the ligating method. Distilled water was administered to the HC and EPC groups and the plant extract was administered to the EP400 and EP800 groups by oral gavage at doses of 400 mg/kg and 800 mg/kg, respectively. The rats were sacrificed on the 15th day. The values of glutathione peroxidase GSH-Px, malondialdehyde (MDA), superoxide dismustase (SOD), interleukin-1β (IL-1β), interleukin-10 (IL-10), total antioxidant status (TAS), total oxidant status (TOS), oxidative stress index (OSI) in the gingival tissues were analyzed by ELISA tests. Alveolar bone loss was assessed using micro-CT images of the maxilla.

**Results:**

Although the IL-1β, TOS, OSI results of the healthy control group were lower than those of the other groups, the TAS values were higher (p<0.05). No significant difference was found in the biochemical parameters among the EPC, EP400, and EP800 groups (p>0.05). Alveolar bone loss was significantly reduced in the extract groups compared to the EPC group (p<0.001).

**Conclusion:**

Within the limitations of this study, it was observed that the systemic *P. eurycarpa* extract application reduced alveolar bone loss in a rat model of experimental periodontitis. Further studies are needed to elucidate the beneficial effects of *P. eurycarpa*.

## Introduction

Periodontal diseases are considered a major public health problem worldwide. Although the aggressive form of the disease affects 11% of the adult population, general periodontal diseases affect more than 50% of the population, making periodontitis one of the most important oral diseases, increasing the global burden of chronic diseases. Periodontitis, of which the main pathogens are *Aggregatibacter actinomycetemcomitans, Porphyromonas gingivalis, Treponema denticola*, and *Tanerella forsythia*, also reduces individuals’ oral health-related quality of life.^1,2^ According to these data, the treatment of periodontitis, which is associated with many systemic diseases, is essential for public health.^3^

Cytokines play a regulatory role in the production and activation of cells with different effects. Inflammatory cytokines increase the bactericidal capacity of phagocytes, recruit additional inherent immune cells to the site of infection, actuate the maturation of dendritic cells, and direct the subsequent immune response to invading microorganisms. Anti-inflammatory cytokines inhibit inflammatory activity or suppress the intensity of inflammation.^4^ Interleukin-1β (IL-1β) exerts a pro-inflammatory effect and plays a role in the pathogenesis of periodontitis.^5^ IL-10, on the other hand, is an anti-inflammatory cytokine and exerts a broad suppressive effect on the production of antigen presenting cells, Th2 cells, Th17 cells, and pro-inflammatory cytokines.^6^ Reactive oxygen species (ROS) are highly reactive oxygen products. At the cellular level, ROS are essential for the physiological processes of eukaryotic cells such as cellular signaling, cellular differentiation, and apoptosis. In addition, ROS play a role in killing pathogens by oxidative means.^7^ Increased ROS formation and decreased antioxidant capacity can increase the destruction of periodontal tissue.^8^ The reaction that occurs when reactive oxygen species affect fatty acids in the cell membrane is called lipid peroxidation. Lipid peroxidation initiates events that can lead to cell death by disrupting the permeability of cell membranes.^9^ Conventional treatment of periodontitis comprises mechanical debridement of microbial plaque and calculus by scaling and root planing. Some chemotherapeutic agents are used to provide additional benefits to the treatment.^10^ Currently, the use of herbal medicines as an adjunctive periodontal treatment is being investigated. It is claimed that herbal products with antimicrobial and anti-inflammatory properties can be beneficial in the control and treatment of periodontitis.^11^

The genus *Pistacia* is a member of the Anacardiaceae family, made up of small trees and shrubs. It belongs to the tropical and subtropical Asian region and has been used by its natives for a long time.^12^ *P. eurycarpa Yalt.* grows especially in the southeastern parts of Turkey.^13^ When the essential oil and chemical contents of the *P. eurycarpa* plant are examined, most of the plant's content consists of α-pinene and β-pinene.^12^ α-pinene and β-pinene are isomers with antibacterial, anti-inflammatory, anticancer, and antifungal effects. As pinenes offer wide safety margins, they can be used with various chemicals.^14,15^

This study aimed to investigate the effect of the *Pistacia Eurycarpa Yalt.* plant extract on alveolar bone loss and the biochemical parameters of gingival tissue in a rat model of experimental periodontitis.

## Methodology

The experimental protocols were designed to comply with animal use ethics and the study was approved by the Local Ethics Committee for Animal Experiments at Inonu University (Confirmation number: 2020/13-4). All experimental procedures were carried out at Inonu University's Experimental Animal Production and Research Center. The biochemical analyses were carried out at Inonu University's Faculty of Medicine, in the Department of Biochemistry.

32 male Wistar albino rats were produced in Inonu University's Experimental Animals Production and Research Center for use in the study. The animals were kept under the same conditions, at a constant room temperature, with a light cycle of 12 hours a day and 12 hours a night. The animals were kept in a plastic cage, each container holding 4 animals. *Ad libitum* nutrition was provided with standard rat chow and water. Rats weighing 200–250 g were included in the study. The rats were randomly divided into 4 equal (n=8) groups: healthy control (HC), experimental periodontitis (EPC), experimental periodontitis + 400 mg/kg plant extract (EP400), and experimental periodontitis + 800 mg/kg plant extract (EP800).

When α=0.05, 1-β(power) = 0.95 and together with the standard deviation values in the alveolar bone loss parameter in the experimental periodontitis study by Kara, et al.^16^ (2013) with grape seed extract, the sample size was calculated as 24. By evaluating the possible losses during the experiment process, it was decided to include 8 rats per group in the experiment.

### Experiment procedure

**Figure 1:**
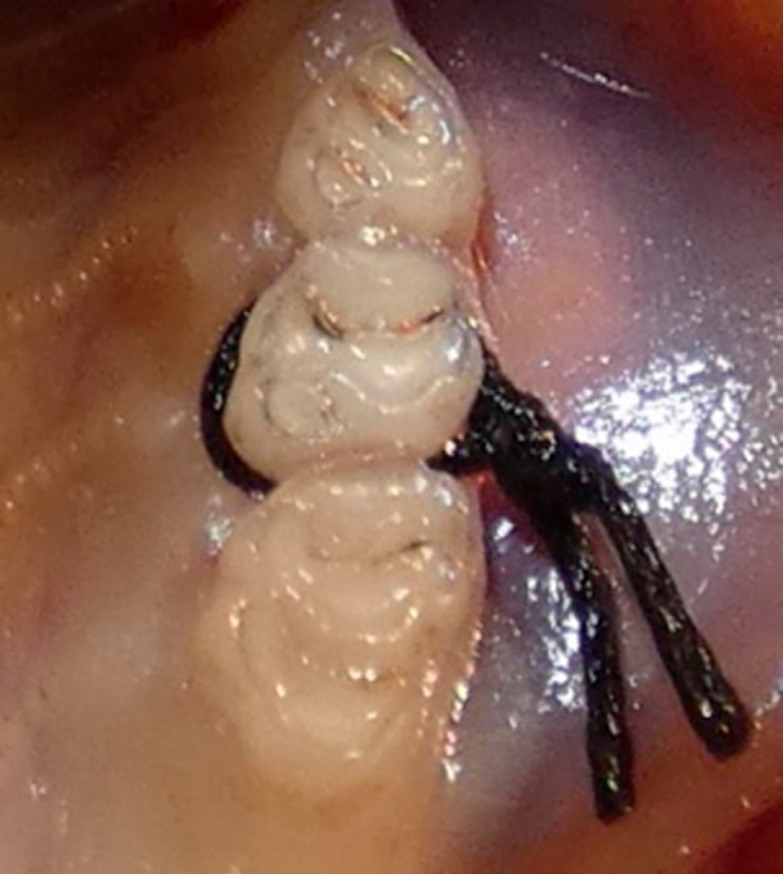
Ligating of the maxillary right second molar

The animals underwent general anesthesia with intraperitoneal administration of ketamine (90 mg/kg) (Doğa İlaç, Turkey) and xylazine (10 mg/kg) (Bioveta, Czech Republic). Then, 4–0 silk sutures (Dogsan, Turkey) were placed subgingivally on the right maxillary second molar in all groups except the HC group (Figure 1). From the first day after suture placement until the 15^th^ day, a single investigator administered distilled water to the HC and EPC groups and *P. eurycarpa* plant extract at doses of 400 and 800 mg/kg to the EP400 and EP800 groups, respectively, by oral gavage at noon. Changes in the animals’ general behavioral patterns were monitored daily. At the end of the experiment, the ligatures were checked and no loss was observed. The animals were sacrificed under high-dose (270 mg/kg ketamine, 30 mg/kg xylazine, ip) anesthesia. The soft tissues around the ligatured teeth were excised, wrapped in aluminum foil, and placed in a deep freezer (Nuire's Glacier, USA) at −80 °C. Maxillectomy was performed using a bone saw disc and maxillary blocks were kept in a 10% formaldehyde solution until micro-computed tomography (micro-CT) images were obtained. The tissue samples and micro-CT images were given randomized numbers and the biochemical analyses and micro-CT calculations were carried out according to these numbers, without the experimental group information.

### Preparation of plant extract

The leaves of the *P. eurycarpa* were collected in eastern Turkey (Malatya) in July and dried in the shade. The dried parts of the plant were ground with a herb grinder. The ground plant material was then macerated in 80% methanol for 24 hours using a mechanical mixer so that the plant's substances could be extracted over a wide solubility range. After 24 hours, the extract was filtered and concentrated in a rotavapor at a temperature not exceeding 45 °C. This maceration and concentration process was repeated 8 times in the same order.^17^ The primary extract obtained at the end of these processes was evaporated in the rotavapor until it no longer condensed and kept in the refrigerator at +4 °C until use. An appropriate amount of plant extract at doses of 400 mg/kg and 800 mg/kg was mixed with 2 ml of distilled water and applied to the animals. This mixture was prepared fresh every day to provide a homogeneous extract.

### Biochemical analysis

The gingival tissues were thawed and phosphate buffer was added to form a 10% homogenate. The tissue homogenates were centrifuged at 4,000 rpm for 25 minutes at +4 °C to obtain supernatants. All biochemical analyses were performed in the supernatant.

Commercially available Rat ELISA kits (Sunredbio Tech. Co. Ltd, Shanghai, China) were used for measurements of IL-1β, IL-10, glutathione peroxidase (GSH-Px), superoxide dismutase (SOD), malondialdehyde (MDA). The tissues were prepared following the kits’ technical guides. The samples obtained for the standard solutions were read with a microplate reader (BioTek Synergy H1, BioTek Instruments, USA) and evaluated with a data analysis program (Gen5, BioTek Instruments, USA). Analysis results were calculated with the calibration curve obtained using standards.

Total oxidant status (TOS) was measured with a commercially available kit (RelAssay Diagnostics, Turkey). The oxidants in the sample oxidize the ferrous ion-chelator complex in the kit, and the potentiator molecules in the reaction medium extend the oxidation reaction.^18^

Total antioxidant status (TAS) was measured with a commercially available kit (RelAssay Diagnostic, Turkey). This kit's principle is based on reducing the colored 2,2’-azino-bis 3-ethylbenzothiazoline-6-sulfonic acid (ABTS) radical included in the kit to form the colorless ABTS by the antioxidants in the sample.^19^ The kit is calibrated with a stable antioxidant stock solution called Trolox equivalent. The oxidative stress index (OSI) values were obtained by dividing the TOS concentrations by TAS.

### Micro-CT measurements

**Figure 2:**
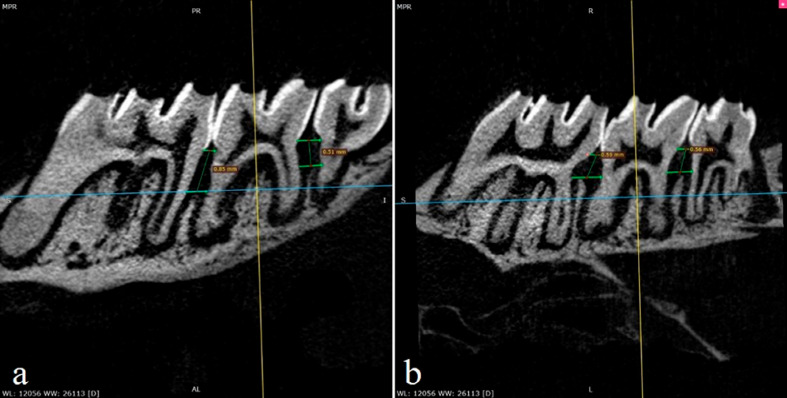
a) Micro-CT measurements on buccal side b) Micro-CT measurements on palatinal side

The maxillary blocks were prepared at a size of 1 cm x 1 cm and the imaging procedures were performed with a micro-CT imaging device (Skyscan 1272, Germany). The images were acquired in DICOM format and analyzed by a single researcher blinded to the groups using the RadiAnt DICOM Viewer (Medixant, Poland) software. Micro-CT measurements were performed as described in the study by Chen, et al.^20^ (2015). Alveolar bone loss was measured by linear measurements using the software's multiplanar reconstruction feature. A line was created between the cemento-enamel junction of the neighboring teeth. Parallel to this line, a second line was created at the first bone in contact with the alveolar bone ridge. Bone loss was determined by measuring the distance between these two lines. The amount of alveolar bone loss in the mesial and distal interproximal region of the second molar tooth was measured as mentioned on the buccal and palatal sides (Figure 2). The measurements were repeated one week later to determine the intraclass examiner agreement. The value of the intraclass correlation coefficient was 0.926. After the measurements were recorded, the means of the mesio-buccal and mesio-palatal measurements and the disto-buccal and disto-palatal measurements were taken to obtain data for the mesial and distal sides.

### Statistical analysis

Statistical analysis was carried out using IBM SPSS Statistics 20 (USA) analysis software. Data were given as mean, standard deviation, median, minimum, maximum, percentage, and number. The Shapiro Wilk-W test was used to evaluate the normal distribution of continuous variables. When comparing continuous variables with more than two independent groups, the Analysis of Variance (ANOVA) test was used if normal distribution was confirmed, and the Kruskal Wallis test was used if it was not. *Post-hoc* Tukey's test was used after the one-way ANOVA test when the variances were homogenous and Tamhane's T2 test was used when the variances were not homogenous. After the Kruskal Wallis test, Kruskal Wallis one-way ANOVA (k samples) test was used for *post-hoc* analysis. Statistical significance level was considered to be p<0.05.

## Results

One rat from the experimental periodontitis group died. The experiment was completed without any further casualties.

### Biochemical results

The tissue levels of IL-1β, IL-10, GSH-Px, MDA, and SOD are shown in Table 1. IL-1β concentrations were higher in all experimental periodontitis groups compared to the healthy control group. However, statistically significant results were found only between the HC and EP400 groups (p<0.05). Although the mean values of the EP400 and EP800 groups were lower than those of the EPC group, there was no statistically significant difference (p>0.05). Tissue concentrations of IL-10 were higher in the EPC group, but no significant difference was found (p>0.05).

**Table 1:** Comparison of IL-1β, IL-10, MDA concentrations and GSH-Px and SOD activities between groups

	HC (n=8)	EPC (n=7)	EP400 (n=8)	EP800 (n=8)	p
	Mean±SD	Mean±SD	Mean±SD	Mean±SD	
IL-1β (pg/L)	5216.00±241.43	5895.97±659.77	5698,96±223,86^a^	5628.11±296.07	0.014*
IL-10 (pg/ml)	428.01±53.93	456.40±37.10	410.40±36.08	404.25±18.32	0.065
GSH-Px (ng/ml)	108.49±21.25	104.37±13.16	103.51±10.86	104.82±9.19	0.906
MDA (nmol/ml)	5.70±0.52	6.37±1.06	5.81±0.67	6.29±0.55	0.197
SOD (ng/ml)	63.83±10.87	57.43±6.65	62.74±3.14	67.97±5.74	0.065

* One-way ANOVA Test (Welch's)

HC: Healthy Control Group, EPC: Experimental periodontitis control group, EP400: Low dose plant extract group, EP800: High dose plant extract group, SD: Standart deviation, IL-1β: interleukin 1-β, IL-10: interleukin 10, GSH-Px: glutathione peroxidase, MDA: malondialdehyde, SOD: superoxide dismutase, p<0.05: Statistically significant,

a Statistically significant in relation to HC

There was no significant difference between tissue GSH-Px activities (p>0.05). The HC group showed the lowest values regarding the tissue concentrations of MDA. It was observed that the activities of the EP400 group were lower than those of the EPC group (p>0.05). There was no significant difference regarding SOD activities measured in the gingival tissue. Although a decrease was observed in the EPC group compared to the EP800 group, it was not statistically significant (p=0.056).

Gingival tissue TAS, TOS concentrations, and OSI values are shown in Table 2. The HC group was significantly different from the EPC and EP groups in terms of TOS concentrations (p<0.05). When TAS concentrations in the tissues were examined, the highest values were observed in the HC group. A significant difference was found between the HC and EP400 groups (p<0.05). When the OSI values obtained by proportioning the TAS and TOS concentrations were examined, a significant difference was found among the HC, EPC, and EP800 groups (p<0.05).

**Table 2:** Comparison of TOS, TAS concentrations and OSI values between groups

	HC (n=8)	EPC (n=7)	EP400 (n=8)	EP800 (n=8)	p
	Mean±SD	Mean±SD	Mean±SD	Mean±SD	
TOS (µmol H_2_O_2_ Equiv/L	7.68±0.80	10.39±1.61^a^	10.82±4.79	12.03±3.08^a^	0.002**
TAS (mmol Trolox Equiv/L)	4.38±0.45^b^	3.74±0.17	3.71±0.60	3.91±0.62	0.050*
OSI (mmol Trolox/mmol H_2_O_2_)	1.78±0.32	2.79±0.47^a^	2.97±1.27	3.09±0.68^a^	0.010*

* One-way ANOVA Test,

** Kruskal Wallis Test

TOS: total oxidant status, TAS: total antioxidant status, OSI: oxidative stress index, p≤0.05,

a Statistically significant in relation to HC,

b statistically significant in relation to EPC

### Micro-CT analysis

**Figure 3:**
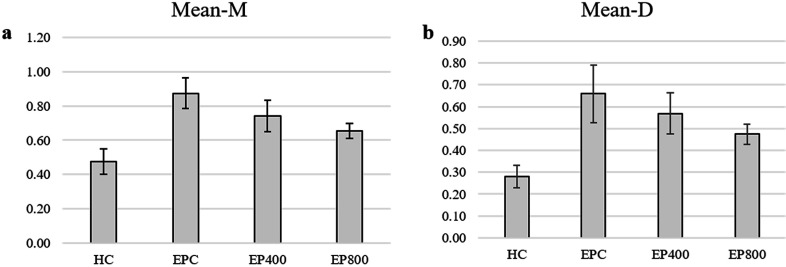
a) Box plot showing mean-mesial (mean-m) values among groups b) Box plot showing mean distal (mean-d) values among groups

The HC group showed statistically significant differences in relation to all the groups in the measurements of alveolar bone loss (Table 3). In addition, the EPC group showed a significant difference in Mean-M values compared to the EP400 and EP800 groups (p<0.05). A statistically significant difference was found between the EPC group and the EP800 group in the Mean-D values (p<0.05) (Figure 3).

**Table 3:** Comparison of alveolar bone loss values between groups

	HC (n=8)	EPC (n=7)	EP400 (n=8)	EP800 (n=8)	p
	Mean±SD	Mean±SD	Mean±SD	Mean±SD	
Mean-M	0.47±0.07	0.87±0.09^a^	0.74±0.09^a^,^b^	0.65±0.05^a^,^b^	0.000*
Mean-D	0.28±0.05	0.66±0.13^a^	0.57±0.09^a^	0.47±0.05^a^,^b^	0.000*

* One-way ANOVA Test

Mean-M: mean mesial alveolar bone loss, Mean-D: mean distal alveolar bone loss, p<0.05,

a Statistically significant in relation to HC,

b Statistically significant in relation to EPC

## Discussion

It has been stated that the use of chemotherapeutic agents may be beneficial, in addition to traditional methods, in the treatment of periodontitis.^21^ On this subject, the use of herbal products, which have proven their effectiveness in the treatment of many diseases and have been reported to cause fewer side effects, is being investigated as an adjunctive treatment for periodontitis.^22^ In previous studies, the *P. Atlantica* plant has been reported to hold antibacterial, antioxidant, and anti-inflammatory effects.^23^ In this study, the extract of *P. eurycarpa*, which is a subspecies of the *P. atlantica* plant, was used. To the best of our knowledge, there are no studies in the literature in which the extract of the *P. eurycarpa* plant has been applied systemically in a rat model of experimental periodontitis . For this reason, studies on the effects and toxic doses of plant extracts from the genus *Pistacia* were taken as a guide regarding the dose to be administered to rats ^24^. As far as we know, the only study on the effect of *P. eurycarpa* on experimental periodontitis is the study carried out by Azeez, et al.^25^ (2020) In this study, Azeez, et al.^25^ (2020) investigated the effect of *P. atlantica kurdica* extract in gel form on osteoclastogenic bone markers in a rat model of experimental periodontitis. When the negative control, positive control, *P. atlantica kurdica* gel, and chlorhexidine gel groups were examined in terms of inflammatory cell counts, RANKL, and IL-1β levels, it was reported that the group applied with *P. eurycarpa* gel showed significantly lower results than the other groups^25^. Arami, et al.^26^ (2014), in their study, compared mouthwash made with the *P. eurycarpa* plant with chlorhexidine mouthwash. It was reported that the lowest values for plaque formation and amount of subgingival microorganism were observed in the *P. eurycarpa* group among the placebo, chlorhexidine mouthwash, and *P. eurycarpa* mouthwash groups.^26^

The role of the host response in periodontal bone loss is complex. Periodontal destruction increases in the presence of insufficient and excessive host response.^27^ Keles, et al.^28^ (2005) reported that the serum IL-1β concentrations of the healthy control group were lower than those of the experimental periodontitis control group in rats. Similar to this study, in our study, it was observed that IL-1β concentrations were low in the HC group. However, no significant difference was found between the EPC, EP400, and EP800 groups. It is possible that the doses of *P. eurycarpa* extract used in the experimental groups did not affect the IL-1β concentrations in the gingival tissue.

IL-10 suppresses the immune system response and the inflammatory response by inhibiting IL-1, IL-6, IL-8, and TNF.^29^ Al-Rasheed, et al.^30^ (2004) reported that bone loss in IL-10-deficient mice increased significantly with age compared to healthy mice. Kurt, et al.^31^ (2019) found that IL-10 concentrations in the control group of experimental periodontitis in rats decreased compared to the healthy group. Kirzioglu, et al.^32^ (2017) reported that IL-10 concentrations decreased in the experimental periodontitis group compared to healthy controls, but it was not statistically significant. In contrast, Silva, et al.^33^ (2017) reported that IL-10 concentrations in the rat serum were elevated in the experimental periodontitis group compared to the healthy group. Although IL-10 concentrations increased in the experimental periodontitis groups in our study, this increase was not statistically significant.

In studies examining the relationship between GSH-Px and periodontitis, Kose, et al.^34^ (2017) reported that although there was a slight increase in GSH-Px activities in the experimental periodontitis group compared to healthy controls, there was no significant difference. Comparing GSH-Px activities in a rat model of experimental periodontitis, Oktay, et al.^35^ (2015) reported an increase in rats with experimental periodontitis compared to healthy controls. In contrast, Yiğit, et al.^36^ (2017) reported a decrease in the experimental periodontitis group. In addition to these data, Tsai, et al.^37^ (2005) stated that the amount of glutathione (GSH) in periodontitis patients was lower than in healthy controls, but there was no significant difference in GSH-Px activities in human serum. The researchers stated that insufficient recycling of GSH after use by antioxidant mechanisms might explain the low GSH levels. GSH-Px activities in our study did not vary significantly between the groups, similar to the data in the study by Köse, et al.^34^ (2017)

Lipid peroxidation (LPO) occurs as a result of the interaction of the cell membrane or lipoproteins with ROS. MDA is the end product of the LPO process^34^. In our study, MDA concentrations in gingival tissue were lowest in the HC group and highest in the EPC group. Similarly, Köse, et al.^34^ (2017) in rat heart tissue and Govindaraj, et al.^38^ (2010) in rat serum reported that MDA concentrations were higher in the experimental periodontitis group compared to the healthy control group.

SOD has been detected in the periodontal ligament in humans and is believed to play an essential role in defense against superoxide secreted by gingival fibroblasts. However, SOD is found predominantly in tissues and SOD activity is low in serum and extracellular fluids. Govindaraj, et al.^39^ (2010) reported that SOD activities were reduced in the experimental periodontitis group compared to the healthy group in their study of experimental periodontitis in rats. Therefore, the fact that SOD, an antioxidant enzyme, was lower in the diseased groups is compatible with these data.

Regarding studies that included TOS, TAS concentrations, and OSI values, in a study in which periodontitis was induced by ligature, it was reported that the TOS concentrations and OSI values of the experimental periodontitis group increased compared to the healthy group. In another study of experimental periodontitis in rats, it was stated that TOS concentrations and OSI values increased and TAS concentrations decreased in the experimental periodontitis group.^40^ Rao, et al.^41^ (2014) reported that TAS concentrations in the serum of periodontitis patients were lower than those of the healthy group in a human study. When the results of our study were evaluated, the lowest TOS concentrations and OSI levels and the highest TAS concentrations were observed in the HC group, which is similar to the results of these studies.

In their study, in which Ma, et al.^42^ (2011) created experimental periodontitis with ligatures in rats, they applied ligatures to different numbers of teeth in the experimental groups to mimic different severity levels of periodontal disease. They examined IL-1β and IL-6 concentrations in rat serum. This study stated that although IL-1β serum concentrations increased in the groups with more ligatures, there was no significant difference. In addition, it was reported that IL-6 levels were significantly elevated in the groups with 3 and 6 ligatures compared to the control group and the group with two ligatures.^42^ In our study, the ligature was applied to only one tooth. This may explain the fact that the biochemical values did not show much variation between the groups in our study.

In terms of alveolar bone loss, values were separated into mesial and distal. The fact that the alveolar bone loss in the experimental periodontitis groups was greater than that in the HC group in terms of Mean-M and Mean-D values indicates that the targeted alveolar bone loss was achieved by ligature induction. In addition, alveolar bone loss in the EP400 and EP800 groups in terms of Mean-M values was lower than in the EPC group and, in terms of Mean-D values, bone loss in the EP800 group was lower than in the EPC group in the plant extract groups, indicating that the application of the plant extract was effective in reducing alveolar bone loss. Although alveolar bone loss values decreased, there was no difference among the EPC, EP400, and EP800 groups in biochemical parameters. It is possible that the doses used in the study were not sufficient to affect these parameters in the gingival tissue. As the tests were only carried out on the gingiva, no information was available about changes in other structures, such as gingival crevicular fluid and serum. Jeong-Hyon, et al.^21^ (2020) examined the use of plant extracts in rat models of periodontitis in their review. The studies included in this review, in which a reduction in alveolar bone loss was observed, also examined parameters such as RANKL/OPG levels, osteoclast count, myeloperoxidase activity, antibacterial effect, osteocalcin level, matrix metalloproteinase expression rate, TNF-α level, and alkaline phosphatase level. Additionally, murine models of periodontitis hold limited translational application in humans. In this context, studies on the use of humanized mice should also be evaluated.^43^ These factors can be considered as limitations of the study. Further studies are required to address these limitations.

## Conclusion

Within the limitations of this study, it was observed that systemic administration of *P. eurycarpa* effectively reduced alveolar bone loss in the rat models of experimental periodontitis. However, no changes were observed in the biochemical parameters examined to understand the mechanism of this effect. In future studies on experimental periodontitis, the use of wider dose ranges, the examination of biochemical parameters in more diverse structures, and the inclusion of other factors affecting periodontal disease in the study may help to uncover the effect mechanism of the *P. eurycarpa* extract.

## Data availability statement

All data generated and analyzed during this study are included in this published article.
